# Electrochemical Impedance Spectroscopy Study of the KI:EG Molar Ratio Effect on the Electrochemical and Interfacial Properties of KI:EG:I_2_ Deep Eutectic Electrolytes for Dye-Sensitized Solar Cells

**DOI:** 10.3390/molecules31071159

**Published:** 2026-03-31

**Authors:** Akotchayé Amenou, Komi Apélété Amou, Essowè Mouzou, Komlan Segbéya Gadedjisso-Tossou, Mazabalo Baneto, Ayayi Claude Ahyi

**Affiliations:** 1 Solar Energy Laboratory, Department of Physics, Faculty of Sciences, University of Lomé, Lomé 01BP 1515, Togobanetopaul@gmail.com (M.B.); 2 Regional Center of Excellence for Electricity Control, University of Lomé, Lomé 01BP 1515, Togo; 3 Physics of Semiconductor Materials and Components Laboratory, Department of Physics, Faculty of Sciences, University of Lomé, Lomé 01BP 1515, Togo; 4 Physics Department, Aubum University, Auburn, AL 36849, USA; ahyiaya@auburn.edu; 5 National Coalition of Independent Scholars, Cambridge, MA 02142, USA

**Keywords:** deep eutectic solvent, potassium iodide, ethylene glycol, molar ratio, electrochemical impedance spectroscopy (EIS), resistance, dye-sensitized solar cells (DSSC), iodine, electrolytes

## Abstract

Using electrochemical impedance spectroscopy (EIS), a technique that analyzes the electrical response of a system subjected to a sinusoidal disturbance in order to probe its physicochemical properties, this study determined an optimal molar ratio of 1:7 between ethylene glycol (EG) and potassium iodide (KI). This composition significantly improves the electrochemical performance of the KI, EG, and I_2_ electrolyte for photovoltaic applications. Four formulations with KI:EG molar ratios of 1:5, 1:7, 1:9, and 1:11 were synthesized. The amount of diiodine (I_2_) was fixed at 0.1 mol% relative to the amount of KI. These electrolytes were then characterized by EIS. The series resistance (Rs), charge transfer resistance (Rct), diffusion resistance (Rw), CPE (constant phase element) parameter, and exponent (n) were extracted and compared. The results show that the formulation with KI:EG = 1:7 has the lowest Rct (3.054 Ω) and Rw (7.296 Ω) values, indicating optimal redox kinetics and improved ion transport within the electrolyte. This molar ratio corresponds to a minimum Rs value (5.612 Ω), indicating reduced series resistance. The mechanisms of solvation, viscosity, and ion diffusion are examined. This work, based exclusively on screening by electrochemical impedance spectroscopy (EIS), highlights the decisive role of solvent composition in electrolyte performance. It identifies an optimal molar ratio window that strikes a balance between redox efficiency and ion mobility, with a view to improving DSSC performance.

## 1. Introduction

The growth in global energy demand, combined with the increase in greenhouse gas emissions, makes it necessary to develop renewable, sustainable, and environmentally friendly energy sources [1]. Among these, solar energy stands out as one of the most promising, given the immense amount of solar radiation reaching Earth, estimated at approximately ${3\times 10}^{24}$ joules per year, which is over $10^{4}$ times the current global energy consumption [2]. Photovoltaic technologies have been developed and enable the direct conversion of solar energy into electricity via the photovoltaic effect in semiconductor materials [3]. A wide range of photovoltaic technologies has been developed over the past decades. Conventional silicon-based solar cells dominate the commercial market, while thin-film technologies rely on materials such as CdTe and CIGS [2]. Emerging technologies, including high-performance perovskite solar cells and organic photovoltaics, have attracted significant attention due to their high-power conversion efficiencies and low-cost fabrication processes [4,5,6]. In parallel, oxide-based semiconductors and ceramic materials have played a crucial role in various solar cell architectures owing to their chemical stability, abundance, and tunable electronic properties. Binary oxide ceramics such as Titanium dioxide ($TiO_{2}$), Zinc oxide (ZnO), Iron(III) oxide (${Fe}_{2}O_{3}$), Tungsten trioxide (W$O_{3}$), Cerium dioxide ($CeO_{2}$), Aluminum oxide (${Al}_{2}O_{3}$), and Silicon dioxide ($SiO_{2}$) have been extensively investigated as photoanodes, electron transport layers, blocking layers, or functional interfacial materials in different photovoltaic systems. A recent comparative and bibliometric analysis published in Ceramics highlights the growing importance and versatility of these oxide materials in solar cell research [7]. A conventional dye-sensitized solar cell (DSSC) consists of four main components: a photoanode, most often made of titanium dioxide (TiO_2_); a photosensitive dye; a counter electrode; and an electrolyte, whose role is to ensure the migration of redox species between the electrodes [8,9,10]. The electrolyte plays a crucial role in regenerating the oxidized dye and transporting charge carriers, particularly iodide $\left(I^{-}\right)$ and triiodide ($I_{3}^{-}$) ions [11]. Conventional dye-sensitized solar cell (DSSC) electrolytes based on organic solvents such as acetonitrile or methoxypropionitrile enable the highest photon conversion efficiencies, up to 14.7% under standard conditions [3,12,13]. However, their practical application is limited by significant drawbacks, including high volatility, substantial flammability, and toxicity, which raise concerns regarding long-term stability and safety.

Deep eutectic solvents (DESs) are a class of alternative solvents formed by combining hydrogen bond donors (HBDs) and hydrogen bond acceptors (HBAs), resulting in eutectic mixtures with significantly reduced melting points [9,10]. Owing to their favorable physicochemical properties, such as high thermal stability, low toxicity, good biodegradability and negligible vapor pressure, DESs have emerged as promising and sustainable alternatives to conventional organic solvents for electrochemical applications [14,15]. The use of DESs as electrolytes in solar cells has the potential to improve open-circuit voltage and short-circuit current, depending on their molecular structure [16]. They therefore represent a promising area of research for increasing both the efficiency and environmental friendliness of photovoltaic technologies. Among the DES systems reported to date, the mixture of potassium iodide (KI) and ethylene glycol (EG) has been identified as a functional deep eutectic solvent and has been explored in several previous studies. In this system, strong hydrogen-bond interactions and ionic interactions govern ion solvation, viscosity and charge transport properties. In particular, Cruz et al. reported the use of alkali iodide-based DES electrolytes (LiI, NaI and KI) combined with ethylene glycol in dye-sensitized solar cells (DSSCs). Among the investigated formulations, the KI:EG electrolyte delivered the best photovoltaic performance, achieving a power conversion efficiency of 2.3% [11,17]. More recently, Xu et al. demonstrated that the KI:EG mixture forms a stable deep eutectic solvent, exhibiting reversible electrochemical redox behavior associated with the iodide/triiodide ($I^{-}/I_{3}^{-})$ couple [17]. This finding clearly confirms the deep eutectic nature of the KI:EG system and highlights its suitability as an electrolyte for electrochemical applications. In the context of DSSCs, KI:EG: I_2_-based electrolytes offer several advantages, including relatively high ionic conductivity, enhanced thermal stability and efficient redox reversibility of the ($I^{-}/I_{3}^{-})$ couple, which facilitates rapid dye regeneration. In this electrolyte system, ethylene glycol acts as a hydrogen bond donor and coordinating solvent, while potassium iodide provides redox-active iodide species and K^+^ cations, which are known to influence interfacial recombination processes and charge transport [11].

Viscosity, a measure of a fluid’s resistance to flow and deformation, is a key property of ionic liquids such as deep eutectic solvents (DESs). It determines the mobility of ionic species [14]. Indeed, high viscosity leads to lower conductivity, and a decrease in viscosity is accompanied by an increase in the number of charge carriers and consequently an improvement in conductivity [18,19]. In DES systems, viscosity and conductivity are strongly dependent on the type and molar ratio of the hydrogen bond donor and acceptor components [14,18]. Despite the growing interest in potassium iodide and ethylene glycol (KI:EG)-based electrolytes, the influence of varying the KI:EG molar ratio on the intrinsic electrochemical behavior and the electrode/electrolyte interfacial properties remains poorly understood. In particular, the link between the composition-induced viscosity of the DES (KI:EG), ionic mobility, and interfacial processes such as charge transfer and redox species diffusion remains poorly understood. Most previous studies have focused primarily on photovoltaic performance at the device level, paying little attention to the underlying interfacial mechanisms.

The main objective of this work is therefore to fill these gaps. Using electrochemical impedance spectroscopy (EIS), a powerful technique that allows for the dissociation of ionic resistance, interfacial processes, and diffusion contributions, this study aims to elucidate the impact of the KI:EG molar ratio on the fundamental properties of the electrolyte. The main results demonstrate that optimizing this ratio is a key lever for modulating viscosity and conductivity, allowing for improved mechanistic understanding prior to integration into the device. So, (KI:EG:I_2_) electrolytes are systematically investigated by varying the KI:EG molar ratio (1:5, 1:7, 1:9 and 1:11), while maintaining a constant iodine content of 0.1 mol% relative to potassium iodide. Electrochemical impedance spectroscopy EIS measurements are performed using a symmetric Pt/electrolyte/Pt configuration, allowing key parameters such as series resistance $R_{S}$, charge-transfer resistance $R_{ct}$ and diffusion-related resistance to be extracted and correlated with electrolyte composition. The selected KI:EG molar ratios cover a relevant physicochemical window for deep eutectic solvents, consistent with ranges reported in the literature for HBD:HBA systems [14,17]. The gradual increase in ethylene glycol content enables systematic trends in ion solvation, viscosity and impedance parameters to be identified. Maintaining a constant iodine concentration ensures sufficiently fast ($I^{-}/I_{3}^{-})$ redox kinetics while avoiding excessive diffusion limitations and recombination losses, thereby isolating the intrinsic effect of the KI:EG molar ratio on the electrochemical impedance response.

## 2. Results

### 2.1. Preparation Results of KI:EG:I_2_ Electrolytes

Four different KI:EG:I_2_ electrolytic formulations, designated E1 to E4, corresponding to molar ratios of 1:5, 1:7, 1:9, and 1:11 respectively, as shown in Table 1, were prepared and characterized by electrochemical impedance spectroscopy (EIS).

**Figure 1:**
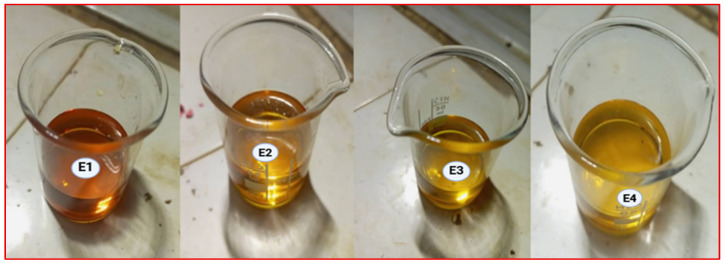
Synthesis results of the electrolytes according to the different molar ratios between KI and EG: E1 (1:5), E2 (1:7), E3 (1:9), E4 (1:11).

The results of the preparation of the electrolytes are presented in that order in Figure 1. The electrolytes exhibit a color ranging from amber to dark brown; the intensity of this color increases with the iodide concentration. This can be attributed to the formation of polyiodide species such as $I_{3}^{-}$ resulting from the equilibrium between molecular iodine $I_{2}$ and iodide ions $I^{-}$ in the KI:EG electrolytic system according to the equation [20]:

$$I_{2}+I^{-}\to I_{3}^{-}$$

### 2.2. Results of Electrochemical Impedance Spectroscopy Characterization of KI:EG:I_2_ Electrolytes (E1, E2, E3, E4)

#### 2.2.1. Nyquist Plot Analysis

**Figure 2:**
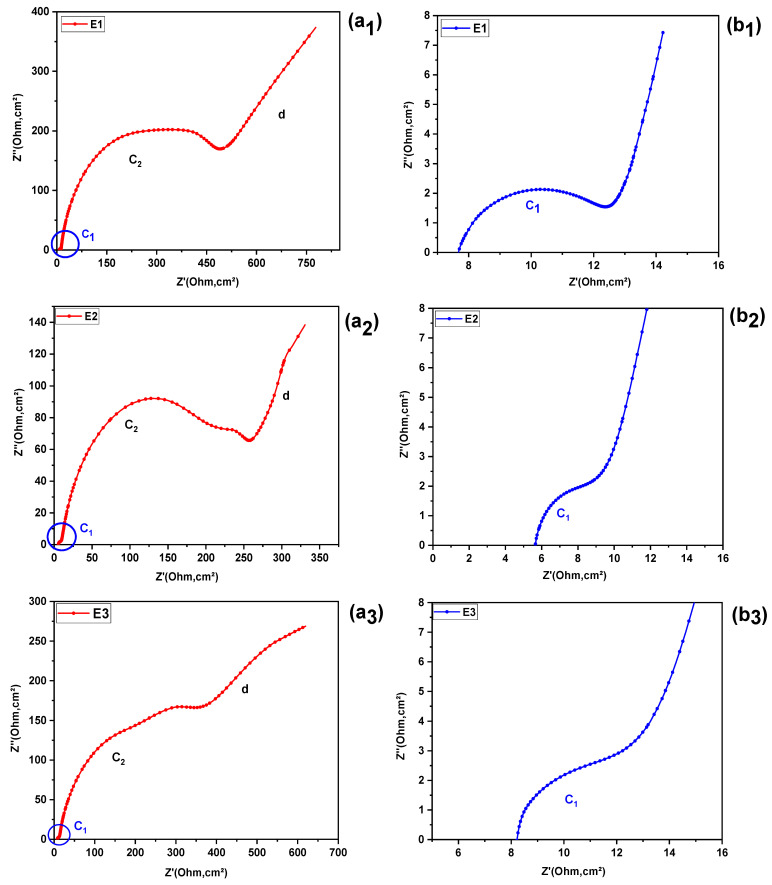

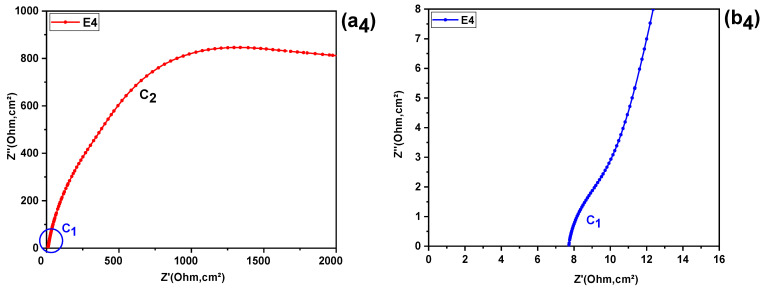
Nyquist plots of E1–E4 electrolytes: $(a_{i}{)}_{i=(1,.\;\ldots ..,4)},$ full spectra and $(b_{i}{)}_{i=(1,.\;\ldots ..,4)}$ enlarged view of the high-frequency region revealing the hidden semicircle. (**a1**) full spectra and (**b1**) enlarged view of the high-frequency region revealing the hidden semicircle for electrolyte E1; (**a2**) full spectra and (**b2**) enlarged view of the high-frequency region revealing the hidden semicircle for electrolyte E2; (**a3**) full spectra and (**b3**) enlarged view of the high-frequency region revealing the hidden semicircle for electrolyte E3; (**a4**) full spectra and (**b4**) enlarged view of the high-frequency region revealing the hidden semicircle for electrolyte.

Figure 2 $(a_{i}$, $b_{i}{)}_{i=(1,.\ldots ..,4)},$ illustrates the results of electrochemical impedance spectroscopy (EIS), presented in the form of a Nyquist diagram. In this diagram, the imaginary part of the impedance (Z″) is plotted as a function of its real component (Z′).

Upon examination of these spectra, it can be seen that each of the four electrolyte formulations analyzed exhibits a distinct semicircular loop. The first arc (C1), appearing at high frequencies (HF), has a very low amplitude and only becomes clearly visible after enlarging this area (Figure 2(b1–b4)). The second arc (C2), observed at intermediate frequencies (IF), is characterized by a significantly larger diameter (Figure 2(a1–a4)).

At low frequencies (LF), however, a pseudo-straight line (d) inclined at approximately 45° can be observed, suggesting diffusive behavior, generally modeled by a Warburg element or by a finite diffusion impedance (Zw).

The shapes of these arcs (flattening and diameter) vary from one electrolyte to another, indicating differences in charge transfer kinetics, interfacial capacitance, and ionic mobility and structure in solution. These variations will be examined in more detail in the Discussion section, where the extracted parameters (Rs, Rct, CPE, diffusion) will be correlated with the physicochemical properties specific to each formulation.

#### 2.2.2. Bode Plot Analysis

**Figure 3:**
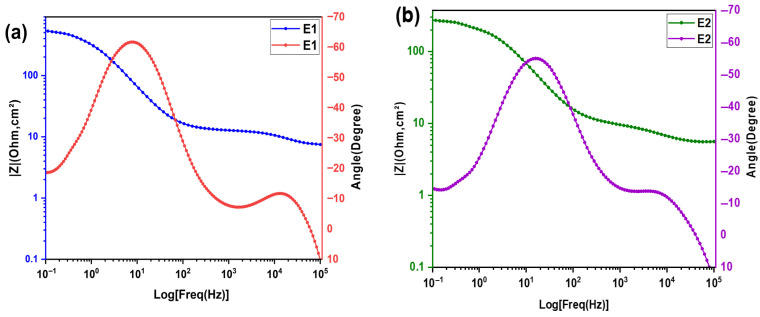

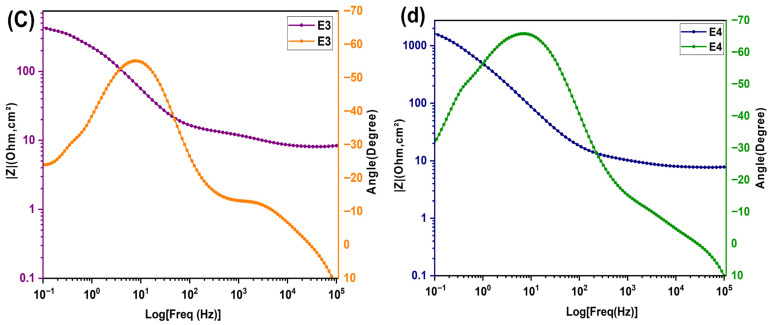
Bode plot for the electrochemical impedance spectra of KI:EG:I_2_ electrolytes at various molar ratios (specifically (KI:EG) = (**a**) E1 (1:5), (**b**) E2 (1:7), (**c**) E3(1:9), and (**d**) E4 (1:11).

The Bode phase diagrams, ranging from E1 to E4, are shown in Figure 3a–d. Each formulation exhibits two distinct phase peaks: The first, at high frequency between ${(10}^{4}-10^{5}\;Hz),\;$ is less pronounced. The second peak, observed at low frequencies ${(10}^{-1}-10^{2}\;Hz)$, is much more pronounced. Regarding the magnitude of Bode, there is a change in slope around the frequencies where the phase peaks are located: the curve evolves from a resistive plateau, characterized by behavior dominated by ohmic resistance, to a decrease that indicates capacitive behavior [21]. These analyses will be correlated with the results of the Nyquist diagrams to identify the electrochemical parameters affected by the composition and determine the optimal electrolyte.

### 2.3. Results of Electrochemical Impedance Spectroscopy Parameter Extraction

**Figure 4:**
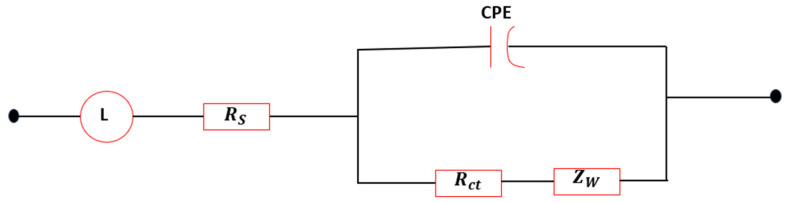
Equivalent circuit model.

**Figure 5:**
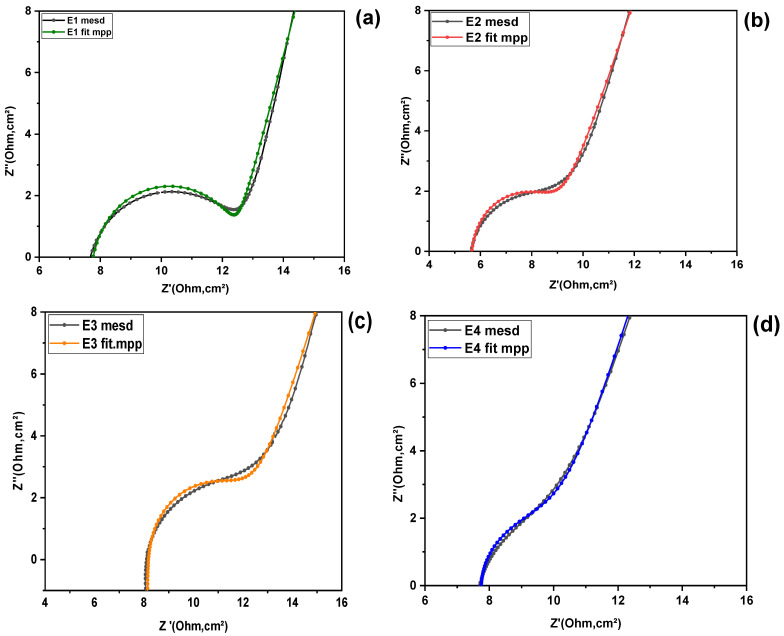
Experimental and fitted Nyquist plots for the electrolyte formulations: (**a**) E1, (**b**) E2, (**c**) E3, and (**d**) E4.

To examine in more detail the influence of the composition of the different KI:EG:I_2_ electrolytic formulations on the electrode reaction kinetics, the electrochemical impedance spectroscopy (EIS) parameters relating to the working electrode/electrolyte interface (as described in the “Parameter Extraction Methodology”section), were determined. These parameters correspond exclusively to the first capacitive semicircular loop (C_1_) in the high-frequency range, limited by a straight line to the low frequencies (scattering, see Figure 2), $(b_{i}{)}_{i=(1,.\;\ldots ..,4)}$, for each electrolyte. The extracted fitting parameters are summarized in Table 2. Figure 4 illustrates the equivalent circuit employed for the impedance analysis, while Figure 5a–d present the corresponding fitting results.

The analysis reveals that the characteristic resistive parameters, namely the solution resistance Rs, the charge-transfer resistance Rct, and the effective diffusion resistance Rw, are lower for the KI:EG = 1:7 formulation compared to the other studied compositions. This indicates improved ionic conductivity and facilitated charge-transfer processes at this specific molar ratio.

In contrast, the capacitive parameters ($Q_{0}$ and the exponent n) associated with the KI:EG =1:7 formulation exhibit higher values, suggesting a more homogeneous and better-structured electric double layer at the electrode/electrolyte interface.

## 3. Quantitative Analysis of Results and Discussion

### 3.1. Effect of KI:EG Molar Ratio on Electrochemical Behavior

**Figure 6:**
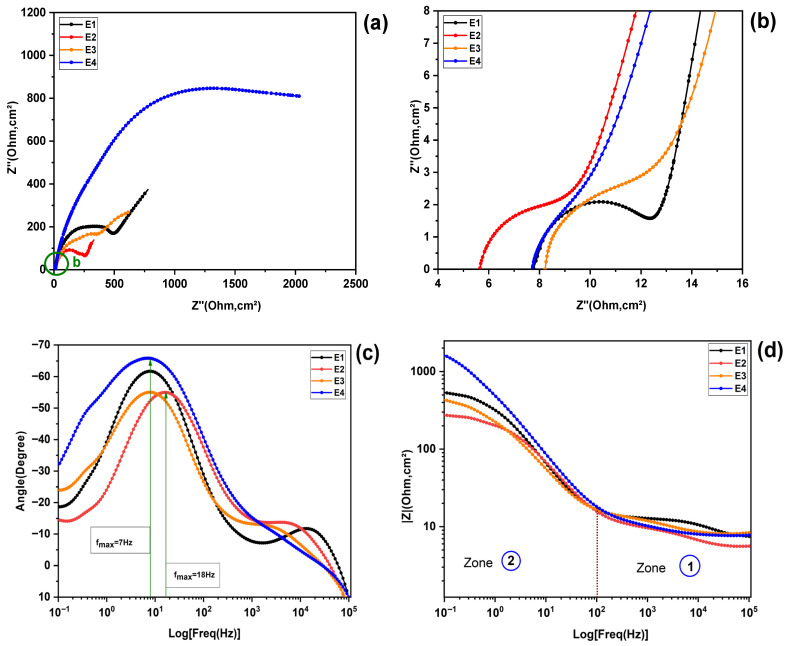
Morphological changes in the Nyquist and Bode plots as a function of the KI:EG molar ratio: (**a**) Full Nyquist spectrum and (**b**) enlarged view of the high-frequency region revealing the hidden semicircle of the electrolytes, (**c**,**d**) Full Bode spectrum.

The morphological changes in the Nyquist and Bode plots as a function of the KI:EG molar ratio are compared for all ratios together and presented in Figure 6 below. Figure 6a,b show the Nyquist diagrams for the four electrolyte formulations combined, while the corresponding Bode diagrams are shown in Figure 6c,d. Three main characteristics can be distinguished for all electrolyte spectra: at high frequency (HF), a depressed arc (Figure 6b) is visible, and an associated peak appears in the high-frequency Bode phase spectrum (Figure 6c); at medium frequency (MF), a second depressed arc, wider in the Nyquist diagram, and another more pronounced peak in the Bode phase spectrum appear (see Figure 6a,c). Finally, a pseudo-straight line appears in the low-frequency region, which is characteristic of Warburg-type behavior [22]. It is important to note that a resistance can be associated with each arc, its value being approximately determined by its diameter [17]. In addition, another resistance (Rs), series resistance, can be identified in the Nyquist spectrum, visible in the enlarged sections, Figure 6b, corresponding to the distance between the origin of the axes and the beginning of the high-frequency arc [23]. The first arc, observed at high frequency, is modeled by a fast charge transfer resistance (Rct) at the platinum electrode and electrolyte interface [24], associated with a constant phase element (CPE), the second, wider arc, observed in the intermediate frequency region, is related to the impedance of the electrolytic solution. This takes into account the mass transport of slower redox species, in particular the redox reaction of iodide $I^{-}$ and triiodide $I_{3}^{-}$ and is modeled by the resistance (R_S_) [22,25]. Finally, the pseudo-slope observed at low frequencies represents a Warburg-type diffusion impedance (Zw/Rw) [21]. By analyzing the variations in the Nyquist arcs, together with the phase peaks and Bode magnitude plots as a function of the KI:EG molar ratio, we aim to identify trends that could suggest a potentially optimal composition within the studied range. This allows for the simultaneous reduction in the charge transfer resistance (Rct), the series resistance Rs (which corresponds to the resistance of the electrolyte), and the diffusion resistance (Rw). This suggests a significant improvement in the electrochemical kinetics throughout the electrochemical system. The diameter of high-frequency Nyquist arcs is closely related to the charge transfer resistance at the electrode-electrolyte interface [17,22]. A smaller arc diameter indicates a reduction in this resistance, which translates into better charge transfer kinetics and, consequently, an improvement in the electrochemical performance of the system. This relationship has been particularly highlighted in recent studies involving deep eutectic solvent (DES) electrolytes. For example, research by Xu and colleagues in 2022 on deep eutectic solvent electrolytes [17], combining KI and EG for supercapacitors, showed that a decrease in the high-frequency arc diameter is associated with a significant reduction in the charge transfer resistance at the electrode-electrolyte interface.

**Figure 7:**
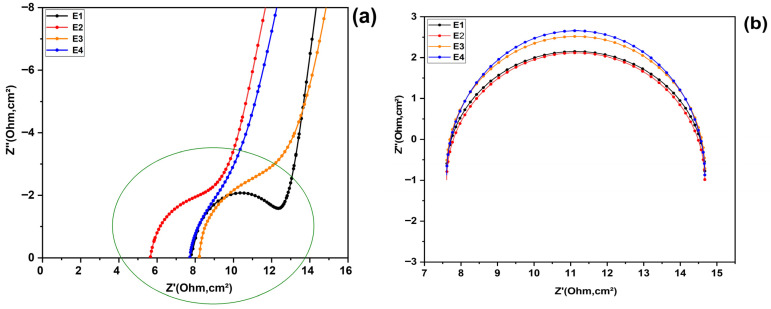

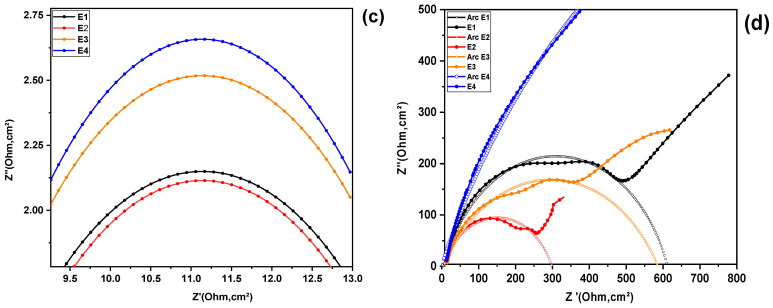
Impedance spectra illustrating the Nyquist arcs associated with the different electrolytes E1, E2, E3 and E4: (**a**) Enlarged view of the high-frequency region revealing the hidden semicircle associated with the electrolyte composition, (**b**) Fitting and comparison of the arcs shown in (**a**), (**c**) Zoomed view of the arc presented in (**b**), (**d**) Comparison of the arcs in the intermediate-frequency region.

Among the four electrolyte formulations studied, electrolyte E2 (KI:EG = 1:7), showed the smallest arc diameter in the high and medium frequency regions on the Nyquist diagram, as shown in Figure 7a–d. Indeed, when we look at D(1:5), D(1:7), D(1:9), and D(1:11), which represent the diameters of the Nyquist circle arcs for the electrolytes E1, E2, E3, and E4 respectively (corresponding to molar ratios of KI:EG of 1:5, 1:7, 1:9, and 1:11), we can observe the following relationship in Figure 7a–d: D(1:7) is less than D(1:9), which is also less than D(1:5), and that is less than D(1:11) in the high- and mid-frequency regions. This response indicates that electrolyte E2 has the lowest charge transfer resistance and lowest mass transfer resistance, which can means that electron transfer at the electrode/electrolyte interface is greatly facilitated and that electron transfer kinetics are optimized at the interface. Indeed, the charge transfer resistance (Rct) is directly linked to the kinetics of electronic exchanges at the electrode/electrolyte interface, particularly for the couple ${(I}^{-}/I_{3}^{-})$. This resistance is correlated with the rate of the electrochemical reaction, which is described by the Butler–Volmer equation. According to this relationship, the exchange current $j_{o}$, which reflects the speed of the electrochemical reaction, depends on the concentration of the redox species and their mobility (Equation (1)) [26].

(1) $$j_{o}=nFk_{o}C_{{I_{3}}^{-}}^{\left(1-\alpha \right)}C_{I^{-}}^{\alpha }$$

In this equation $C_{{I_{3}}^{-}}\;and\;C_{I^{-}}$ denote the concentrations of triiodide and iodide, respectively, $k_{o}$ is the rate constant, α represents the electron transfer coefficient, n is the number of electrons involved, and *F* is Faraday’s constant. It is important to note that the transfer resistance ($R_{ct}$) is inversely proportional to this exchange current (Equation (2)) [26].

(2) $$R_{ct}=\frac{RT}{nFj_{o}}$$

The enhanced electrochemical response of E2 could therefore be attributed, at least partially, to its particular composition. In fact, the viscosity of a deep eutectic solvent (DES) depends on the nature and molar ratio of the hydrogen bond donor (HBD) and hydrogen bond acceptor (HBA), as well as on the intermolecular interactions between them.The stronger the interactions, such as hydrogen bonds or van der waals force, the higher the viscosity of the DES [14]. In this case, the DES (E2) formulation allows both an optimal concentration of the redox couple $I^{-}/I_{3}^{-}$ to be achieved and a lower viscosity than that of the other electrolytes (E1, E3, E4) to be obtained. This lower viscosity improves ionic mobility, ensuring that a greater quantity of ions are available for the electrochemical reaction. In addition, it promotes better ion mobility and more efficient solvation thanks to ethylene glycol, thereby reducing energy barriers at the electrode/electrolyte interface. All of these factors contribute to improving the reaction kinetics of the $I^{-}/I_{3}^{-}$ couple, which results in a low charge transfer resistance Rct value.These results are consistent with the work of Hauch and Georg, who revealed that the type of solvent and cation has a significant impact on interfacial resistance. In particular, small cations such as Li^+^ allow for more efficient charge transfer at the interface between the electrode and the electrolyte, compared to larger cations such as tetrapropylammonium (TPA^+^). For the latter, two mechanisms of electron transfer at the interface can be considered: the desolvation of ions followed by their adsorption on the electrode, and charge transfer in the solvated state via a tunneling effect through a solvation layer [26].

The strong dependence of charge transfer resistance on the solvent could therefore be related to its viscosity in the first mechanism, or to the size of the solvent molecules in the second. In the study in question, ethylene glycol, being a hydrogen bond donor, could influence these mechanisms by affecting solvation and ionic mobility in the electrolytic medium [27]. Indeed, an excess of ethylene glycol often leads to an increase in the viscosity of the solution, which slows down the diffusion of redox species (I^−^/I_3_^−^) and limits electron transport, thus leading to a significant increase in charge transfer resistance [17]. In addition, a solvent that is too rich in ethylene glycol promotes the formation of solvated aggregates around iodide and triiodide ions, reducing their availability for redox reactions. Conversely, an insufficient amount of ethylene glycol compromises the stability of the eutectic complex (KI:EG:I_2_), which can reduce the overall conductivity of the electrolyte. It is therefore essential to find an optimal balance in the concentration of ethylene glycol. Too much EG can increase viscosity and slow down transport, while too little EG can weaken solvation and encourage losses through recombination [14,28].

The Bode phase curves for formulations E1 to E4 are shown in Figure 3a–d. The frequence when the phase angle reaches its maximum is inversely proportional to the characteristic relaxation time [17,22].

(3) $$\tau =\frac{1}{f_{max}}$$

which is related to charge transfer processes. The Bode curve for formulation (E2) reveals a phase angle peak, with a significant frequency shift around 18 Hz (see Figure 6c). This indicates a shorter relaxation time, which promotes faster charge transfer than for the other formulations. This behavior supports the results of the Nyquist analysis, confirming that formulation E2 offers the most promising electrochemical performance among those tested.

**Figure 8:**
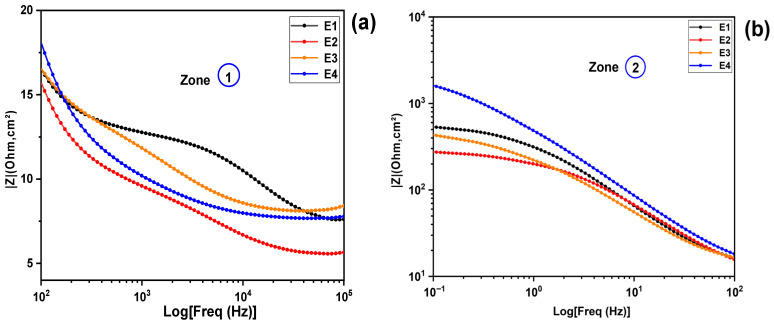
Impedance behavior as a function of frequency for electrolytes E1–E4 with different KI:EG molar ratios: (**a**) high-frequency region, (**b**) low-frequency region.

The results obtained from the Nyquist spectrum diagram are corroborated by the complementary analysis of the Bode magnitude diagrams, as illustrated in Figure 6d. Figure 6d provides an overview of the electrochemical impedance (|Z|) behavior as a function of frequency for several electrolytes, including E1, E2, E3, and E4. The frequencies studied range from 100 KHz to 0.1 Hz. In Figure 6d, two distinct zones (1 and 2) are highlighted, corresponding to the high- and low-frequency ranges, respectively. To allow a more detailed analysis and discussion of the electrochemical behavior of the studied electrolytes, each zone has been enlarged and is presented in Figure 8a,b below. This classification by frequency ranges allows for a better comparison of the behavior of each electrolyte in the ohmic (high-frequency); interfacial and diffusive (low-frequency) domains. At high frequencies $\;{(10}^{2}-10^{5}\;Hz),$ corresponding to the circled area (1) in Figure 6d and Figure 8a, by analyzing the impedance, denoted as Z, for each electrolyte, we observe that Z(E2) is lower than Z(E4), which is itself lower than Z(E3) and finally Z(E1). At low frequencies ${(10}^{-1}-10^{2}\;Hz)$, corresponding to the circled area (2) in Figure 6d and Figure 8b, which are influenced by diffusion, recombination phenomena and charge transfer, a clear order emerges for the overall impedance of each electrolyte: Z(E2) < Z(E3) < Z(E1) < Z(E4) (see Figure 8b). Here, electrolyte E2 stands out for its lower impedance, indicating relatively low charge transfer resistance (Rct), electrolytes resistance (R_S_) and diffusion resistance (Rw). In contrast, E1 and especially E4 exhibit high impedance at low frequencies, indicating that charge transfer processes at the interfaces are slower.

### 3.2. Comparison of the Parameters Derived from the Study of Reactions at the Electrode/Electrolyte Interface for Electrolytes E1, E2, E3 and E4 as a Function of Molar Ratios

**Figure 9:**
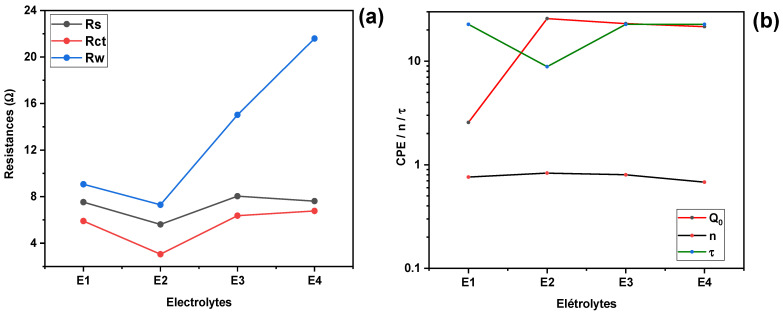
Evolution of electrochemical parameters as a function of the KI:EG molar ratio for (**a**) resistances (Rs, Rct, and Rw) and (**b**) Constant Phase Element (CPE) parameters ($Q_{o}$ and n) and relaxation time (τ).

The analysis of electrochemical resistances, including Rs (series resistance), Rct (charge transfer resistance), and Rw (diffusion resistance), as a function of the K:EG molar ratio reveals interesting trends. The series resistance Rs is observed to decrease from a value of (7.526 Ω) for a KI:EG molar ratio of 1:5, reaching a minimum of (5.612 Ω) for a ratio of 1:7, before increasing for ratios of 1:9 and 1:11 (as presented in Figure 8a). This minimum of Rs at the 1:7 molar ratio indicates that this ratio appears to favor better ion solvation and increased mobility in the KI:EG mixture, probably due to an optimal balance between the free ion concentration and the viscosity of the system. As for the charge transfer resistance (Rct), it is closely related to the kinetics of electronic exchanges at the interface between the electrode and the electrolyte, in particular for the ${(I}^{-}/I_{3}^{-})$, couple [25]. The data from Table 2 and Figure 9a show that transfer resistance (Rct), also decreases, from (5.903 Ω) for a ratio of 1:5 to a minimum value of (3.054 Ω) at the ratio of 1:7. A low Rct is synonymous with a high-exchange current, which promotes rapid regeneration of the dye [27,29], and can improve the performance of the solar cell. The fact that Rct reaches its minimum for the 1:7 ratio confirms that this ratio allows a good balance between ionic conductivity and redox kinetics at the electrode/electrolyte interface, thus facilitating the achievement of an optimal triiodide ion ($I_{3}^{-}$) concentration. This observation aligns with the Butler–Volmer theory, which states that the exchange current $J_{o}$ is proportional to the concentration of redox species, especially ($I_{3}^{-}$) and inversely proportional to Rct, Equations (1) and (2). The reduction in charge transfer resistance observed at the 1:7 molar ratio could be attributed to an optimal $I_{3}^{-}$ concentration and an adequate viscosity of the KI:EG solvent, which stimulates ion mobility. Previous studies have also shown that solvent viscosity has a significant impact on charge transfer resistance at the electrode/electrolyte interface. Low viscosity facilitates ion dissociation and movement, thus reducing resistance to electron transfer [9]. Diffusion resistance (Rw), which concerns the diffusion of ionic species, mainly $I_{3}^{-}$ in the electrolyte, is also strongly influenced by the molar ratio. It decreases by 9.064 Ω and reaches a minimum of 7.296 Ω for a molar ratio of 1:7, before increasing for ratios of 1:9 and 1:11. This increase in Rw at ratios of 1:9 and 1:11 suggests increased limitations to mass transport, probably due to high viscosity that hinders the diffusion of ionic species, the possible formation of ion-solvent complexes that hinder the mobility of charge carriers, and excessive dilution of active ions in the medium. Indeed, the diffusibility D is related to the viscosity η of the solvent (Equation (4)), according to the Stokes–Einstein law [9].

(4) $$D=\frac{K_{B}T}{6\pi \eta r}$$

with $K_{B}$ representing the Boltzmann constant, T the absolute temperature, and r the hydrodynamic radius of the ion in solution. Thus, the diffusion resistance Rw is inversely proportional to the diffusion coefficient D, which can be summarized as (Equation (5)):

(5) $$R_{w}\propto \frac{1}{D}$$

therefore, if the viscosity (η) increases, the diffusion coefficient (D) decreases, leading to an increase in (Rw). The electrolyte E2, corresponding to a molar ratio of KI:EG (1:7), is distinguished by its low diffusion resistance Rw, which indicates a high diffusion coefficient, excellent conductivity, and efficient circulation of ionic species. These characteristics are particularly favorable for the operation of dye-sensitized solar cells. Figure 9b shows that both the CPE parameter $Q_{0}$ and the exponent n increase with the KI:EG molar ratio, reaching their maximum values at 1:7. Beyond this ratio, both parameters gradually decrease. The 1:7 composition, which exhibits the highest $Q_{0}$ and n values, indicates a more capacitive and homogeneous electric double layer. This suggests improved electrode surface coverage and a more uniform adsorption of electroactive species at the platinum/electrolyte interface [14,30]. The exponent n varies between 0.68 and 0.83, reflecting the non-ideal capacitive behavior of the platinum/electrolyte interface. Values closer to unity (0.83) indicate a relatively well-organized double layer, corresponding to a more homogeneous interface and efficient adsorption of $I_{3}^{-}$ ions onto the platinum electrode. In contrast, lower values (0.68) reveal increased interfacial heterogeneity, which may arise from partial surface coverage by adsorbed species, surface irregularities, or a broader distribution of relaxation times associated with charge transfer and ion transport processes.

The evolution of n with the KI:EG molar ratio confirms that electrolyte composition strongly influences interfacial structuring. This behavior is consistent with the progressive attenuation of the high-frequency phase peak and the corresponding modifications observed in the Nyquist semicircles.

Figure 9b also shows that the relaxation time τ, reaches a minimum value of 8.85 ms for the electrolyte with a molar ratio of 1:7, indicating faster charge transfer kinetics at the electrode/electrolyte interface [6,9]. These results identify E2 as the most favorable formulation, simultaneously minimizing all three resistive components. The lower Rs observed in E2 indicates superior bulk ionic conductivity, which can be attributed to optimal ion solvation and mobility in the DES matrix. An excess of KI (as in E1) may lead to increased viscosity and ion pairing, which hinders ion mobility. On the other hand, an excess of EG (as in E4) can dilute the ionic species too much, reducing the number of available charge carriers and increasing both Rct and Rw. The reduction in Rct with E2 reflects more efficient electron exchange at the electrode interface, likely due to better triiodide regeneration kinetics and favorable ion interactions at the Pt surface. Furthermore, lower Rw values correspond to faster diffusion of I^−^/I_3_^−^ species, essential for sustaining high photocurrent in DSSC operation [9,14,26]. These findings confirm the strong influence of the KI:EG molar ratio on electrolyte performance and reinforce the importance of compositional tuning in DES development for energy applications.

## 4. Literature Correlation

The results obtained are consistent with the literature on deep eutectic solvents based on choline chloride/urea, where the electrochemical properties are governed by the balance between ionic strength, viscosity, and hydrogen bonding network. Ethylene glycol ensures the stabilization of I_3_^−^ species while maintaining a viscosity favorable to ionic diffusion. This effect is optimal for intermediate molar ratios, particularly KI:EG ratios of 1:7, for which ionic dissociation is maximal without overdilution of the redox species. The beneficial role of (K^+^) ions on interfacial properties also contributes to the improved electrochemical performance observed for the E2 electrolytic formulation. EIS analysis shows that, for a constant amount of diiodine equal to 0.1 mol% relative to that of KI, the electrolytic formulation KI:EG = 1:7 exhibits minimal series resistance values of series resistance $R_{s}\;$(5.612 Ω), transfer resistance $R_{ct}\;$(3.054 Ω) and diffusion resistance $R_{W}\;$(7.296 Ω) respectively, reflecting increased ionic conductivity, improved charge transfer kinetics at the Pt/electrolyte interface, and efficient diffusive transport of ($I^{-}/I_{3}^{-}$) species. Formulations deviating from this optimal ratio show a progressive degradation of EIS parameters, indicating that an excess of solvent or salt is detrimental. These results confirm the need for precise optimization of the KI:EG ratio, even at constant redox content, for the development of high-performance DES electrolytes for photovoltaic applications.

## 5. Materials and Methods

### 5.1. Electrolytes Preparation

All chemicals used were of analytical grade and utilized without further purification. Potassium iodide (KI, ≥99.5%), ethylene glycol (EG, ≥99%), and iodine (I_2_, ≥99%) were sourced from Sigma Aldrich (St. Louis, MO, USA). KI was selected as the redox-active salt providing iodide $I^{-}$ species. At the same time, EG served as the hydrogen bond donor to form a deep eutectic system with KI, as described in recent studies on DES-based electrolytes [11,17]. Iodine was added to introduce the I^−^/I_3_^−^ redox couple essential for dye regeneration [11]. Reagents were stored in the dark and in dry conditions to preserve their integrity.

The equipment included an analytical balance (RADWAG Wagi, Radom, Poland, model PS210.R2, precision: 0.001 g), a heating magnetic stirrer (IKA C-MAG HS 7, Staufen, Germany, max. temperature: 550 °C), and a potentiostat/Galvanostat CS350M from Wuhan corrtest instruments corps, Ltd. (Wuhan, China).

The electrolytes were prepared by fixing the amount of potassium iodide (KI) as a reference component. The molar amounts of diiodine (I_2_) and ethylene glycol (EG) were determined relative to that of potassium iodide (KI), using the molar ratios targeted for this study. The molar amount of potassium iodide was calculated from the following relationship (Equation (6))

(6) $$n_{KI}=\frac{m_{KI}}{M_{KI}}$$

$m_{KI}\;(15.275\;g)\;$ is the mass of potassium iodide (KI) weighed out and $M_{KI}$ is its molar mass. The amount of substance of I_2_ was fixed at 0.1 mol % relative to that of (KI) (Equation (7))

(7) $$n_{I_{2}}=x\times \;n\left(KI\right)$$

then (Equation (8))

(8) $$m_{I_{2}}=n_{I_{2}}\times M_{I_{2}}$$

with x = 0.001 (0.1%)

Ethylene glycol was added according to the target KI:EG molar ratio. The amount of ethylene glycol to be added was determined using the target molar ratios (KI:EG) for this study. The general relationship used to determine the amount of ethylene glycol is:

(9) $$n_{EG}=r\;\times \;\;n\left(KI\right)$$

where (r) represents the change in the molar ratio of (EG) relative to that of KI (Equation (10)).

(10) $$m_{E_{G}}=n_{E_{G}}\times M_{E_{G}}\;$$

**Figure 10:**
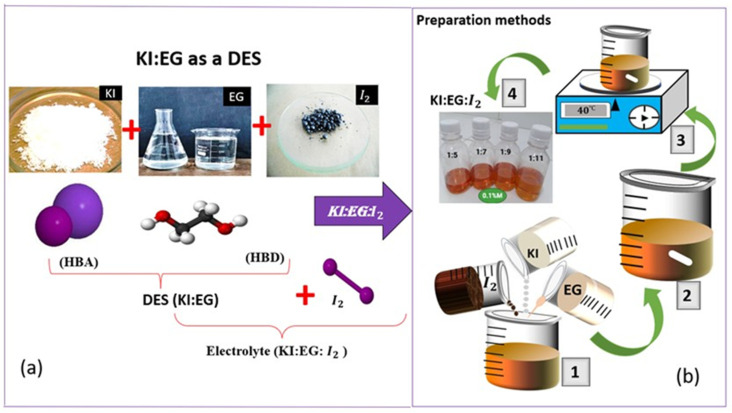
(**a**) A schematic illustration of the formation of the deep eutectic solvent KI:EG (DES) [17]; (**b**) experimental details of the electrolyte (KI:EG:I_2_) synthesis: (1) mixing of potassium iodide (KI), iodine (I_2_) and ethylene glycol (EG); (2) introduction of the magnetic stir bar; (3) magnetic stirring at 40 °C for 60 min; (4) electrolyte KI:EG:I_2_.

Figure 10a shows a schematic illustration of the formation of the deep eutectic solvent KI:EG (DES), where potassium iodide (KI) acts as a hydrogen bond acceptor and ethylene glycol (EG) as a hydrogen bond donor. The subsequent addition of diiodine leads to the formation of the redox electrolyte KI:EG:I_2_. The schematic representation of the KI:EG:I_2_ electrolyte preparation protocol, as described by previous studies [14], is shown in Figure 10b. As shown in Figure 10b, after calculated and weighing the appropriate masses of the constituents (KI, EG, and I_2_), as described above Equations (6)–(10), they are mixed to obtain a mixture (1). A magnetic stir bar is then introduced into the mixture (2), which is subjected to magnetic stirring at 40 °C for 60 min, resulting in a homogeneous solution (3). This homogeneous mixture solution constitutes the electrolyte KI:EG:I_2_ (4).

### 5.2. Electrochemical Impedance Spectroscopy (EIS) Characterization of Electrolytes E1–E4

EIS characterization was performed using potentiostat/Galvanostat CS350M from Wuhan corrtest instruments corps, Ltd. (Wuhan, China),frequency response analyzer. Electrochemical impedance spectroscopy (EIS) measurements were carried out in a standard three-electrode configuration:

- Working electrode (WE): platinum (Pt);
- Counter electrode (CE): platinum (Pt);
- Reference electrode: Ag/AgCl.

**Figure 11:**
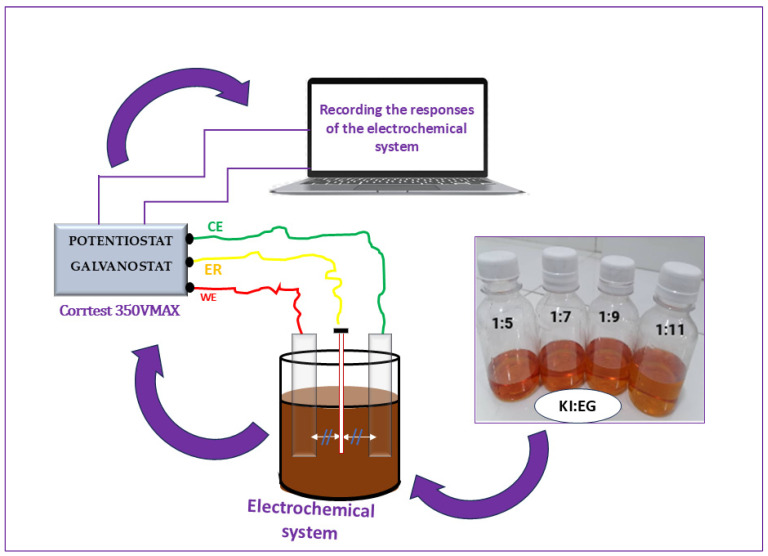
Schematic representation of the electrochemical impedance spectroscopy (EIS) measurement setup for electrolyte characterization.

The Ag/AgCl reference electrode was positioned between the working electrode (WE) and the counter electrode (CE), at a distance of approximately 1 cm from each. It contains a saturated KCl standard solution (3M). This reference electrode is isolated from the KI:EG:I_2_ electrolyte to prevent contamination, while maintaining ionic contact with the solution, thus ensuring accurate and stable measurement of the working electrode potential. A schematic representation of the different stages of the electrode/electrolyte interface characterization is shown in Figure 11. The EIS measurements were performed over the frequency range of 100 KHz to 0.1 Hz by applying a 10 mV AC disturbance at a temperature of 23 ± 2 °C. The temperature was monitored with an ambient thermometer but was not actively controlled. The two platinum (Pt) electrodes, each measuring 2 cm × 2 cm, were immersed in the electrolyte using crocodile clips. The immersed portion of each electrode was 1.5 cm, resulting in an active surface area of 1.5 cm × 2 cm = 3 cm^2^ per electrode. The electrodes were arranged in a parallel-plate configuration, facing each other with a constant inter-electrode spacing (WE-CE) of approximately 2 cm maintained for all measurements. the cell has a cylindrical geometry as shown in Figure 11.

The response of the electrolytic system (KI:EG:I_2_) to the disturbance is obtained as an impedance spectrum.

### 5.3. Electrochemical Impedance Spectroscopy Parameter Extraction

**Figure 12:**
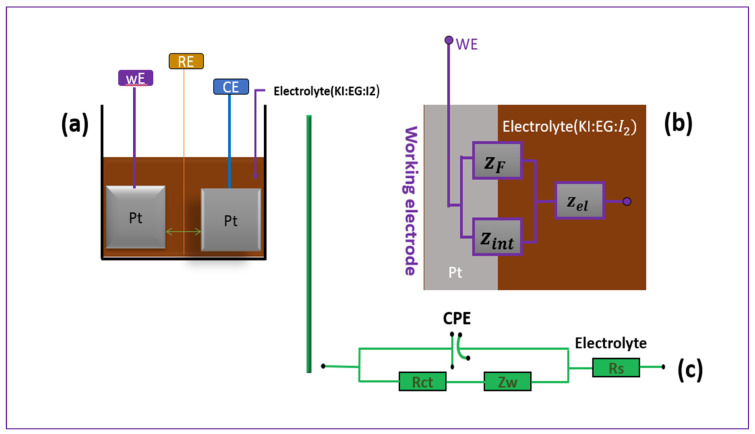
This figure illustrates the experimental setup along with the equivalent model used for Electrochemical Impedance Spectroscopy (EIS) analysis. In part (**a**), the electrochemical system featuring three electrodes is presented—the working electrode (WE), reference electrode (RE), and counter electrode (CE)—all immersed in the electrolyte solution composed of potassium iodide (KI), ethylene glycol (EG), and iodine (I_2_). Part (**b**) presents a physical representation of the interface between the electrode and electrolyte, which serves as a conceptual model for impedance. Finally, part (**c**) displays the equivalent circuit used in the EIS.

With the main objective of this work being the optimization of the electrode/electrolyte interface, the interfacial parameters were determined and analyzed as a function of the composition of the different electrolytic formulations (KI:EG:I_2_). To this end, the interface between the working electrode and the electrolyte was modeled using an equivalent electrical circuit approach, based on the work of Aliaksandr S. Bandarenka and his collaborators (2013) [25]. Figure 12a presents the electrochemical system featuring three electrodes and Figure 12b the different interfacial parameters considered in this modeling namely ($Z_{F},Z_{\mathrm{int}})$. Due to the three-electrode configuration and the presence of the $\left(I^{-}/I_{3}^{-}\right)$ redox couple, the electrochemical response at the electrode/electrolyte interface is dominated by a faradaic process, represented by a general impedance $Z_{F}$, which integrates both the kinetics of the redox reaction and the diffusion of electroactive species towards the surface of the working electrode. Thus, the faradaic impedance can be decomposed into two contributions: $Z_{F}=R_{\mathrm{ct}}+Z_{W}$, where $R_{ct}$ is the charge transfer resistance and $Z_{W}$ is the Warburg diffusion impedance. The response of the electrical double layer at the electrode/electrolyte interface is represented by the interfacial impedance $Z_{int}$, modeled by a constant phase element (CPE) to account for the non-ideal capacitive behavior of the interface. Finally, the resistance of the volumetric electrolyte located between the working electrode and the reference electrode is represented by the impedance $Z_{\mathrm{el}}$, approximated by an ohmic resistance $R_{S}.$

The corresponding equivalent electrical circuit is shown in Figure 4. It consists of two elements arranged in series: on the one hand, the solution resistance $R_{s}$, and on the other hand, a parallel branch comprising the CPE associated with the electrical double layer, as well as the charge transfer resistance $R_{\mathrm{ct}}$ and the diffusion impedance $Z_{W}$, representing the impedance associated with mass transport limitations of redox species toward the electrode interface.

(11) $$Z_{\mathrm{total}}=R_{s}+Z_{\;\mathrm{parallel}}$$

The equivalent impedance of the two elements in parallel is:

(12) $$\frac{1}{Z_{\mathrm{parallel}}\left(w\right)}=\frac{1}{Z_{\mathrm{CPE}}}+\frac{1}{R_{\mathrm{ct}}\;+Z_{w}}$$

with Rw (see Table 2), corresponds to the resistive component of a finite-length Warburg element obtained from the *W*d2 = $Z\left(w\right)$ fitting.

(13) $$Z_{\mathrm{CPE}}\;(w)=\frac{1}{Q_{0}({\mathrm{jw})}^{n}}$$

where (0 < n ≤ 1). If *n* = 1, the CPE behaves like a perfect capacitor with *Q*_0_ = *C* [22].

The total impedance of the system, which includes the electrolyte resistance $R_{s}$ in series with the interface impedance, is given by (Equation (14)).

(14) $$Z_{\mathrm{total}}=R_{s}+\frac{R_{\mathrm{ct}}+Z_{W}}{1+Q_{0}({\mathrm{jw})}^{n}(R_{\mathrm{ct}}+Z_{w})}$$

Spectral fitting and parameter extraction from (Equation (14)) were performed using the freeware EC-Lab (BioLogic Science Instruments). We used the Z-Fit function to adjust the Nyquist curves. The extracted values, including series resistance (Rs), charge transfer resistance (*R*ct), diffusion resistance (*R*w), as well as the CPE (constant phase element:${\;Q}_{0}$ parameter and exponent (*n*), are summarized in Table 2 and illustrated in Figure 9 above.

## 6. Conclusions

This study explored the influence of the KI:EG molar ratio on the electrochemical properties of deep eutectic electrolytes for dye-sensitized solar cells (DSSCs), with the amount of diiodine kept constant at 0.1 mol%. It was carried out by systematic three-electrode EIS analysis of four different formulations (E1 to E4), corresponding to molar ratios of KI:EG of 1:5, 1:7, 1:9, and 1:11. Electrochemical impedance spectroscopy (EIS) was used to evaluate key parameters such as series resistance (Rs), charge transfer resistance (Rct), and diffusion resistance (Rw). The results revealed a nonlinear dependence of electrochemical performance on the KI:EG ratio. Among all formulations, the 1:7 molar ratio (formulation E2) provided the most favorable electrochemical characteristics, exhibiting the lowest values of Rs, Rct, and Rw. This formulation showed a smaller Nyquist semicircle, indicating more efficient electron transfer at the electrode/electrolyte interface, as well as a shorter relaxation time in the Bode diagram, confirming faster redox kinetics. These improvements can be attributed to an optimal balance between ionic concentration and medium viscosity, promoting better solvation and mobility of ionic species. The results also highlight the sensitivity of the electrochemical interface to the structure and composition of the solvent. An excess of KI led to increased viscosity and possibly ion pairing, while an excess of EG diluted the active redox species, which had a negative effect on charge transfer and diffusion. The 1:7 molar ratio appears to minimize these limitations by combining sufficient ionic strength with a favorable solvation environment. From a practical standpoint, these results suggest that the 1:7 molar ratio is an optimal electrolyte formulation that can improve the overall performance of DSSCs, particularly by promoting reduced ohmic losses and faster redox kinetics, which could translate into increased short-circuit current (Jsc) and form factor (FF) in actual devices. Overall, this work highlights the critical role of electrolyte formulation in DSSC performance and provides evidence that the KI:EG molar ratio is a determining factor in achieving optimal charge transport conditions. However, it is important to note that the conclusions presented here are based on electrochemical screening performed by electrochemical impedance spectroscopy (EIS) in a standard three-electrode configuration. Future work will focus on integrating these formulations into complete DSSC devices, followed by characterization under standard illumination (AM 1.5G) in order to directly evaluate the photovoltaic parameters (Jsc, Voc, FF, and efficiency). Additional studies on long-term stability will also be necessary to confirm the practical viability of this DES-based electrolyte. This information serves as a basis for the rational design of DES-based electrolytes and paves the way for future research on photoelectrochemical performance under illumination and long-term stability testing.

## Figures and Tables

**Table 1:** Nomenclature of the results.

$n\left(I_{2}\right)$= 0.1 mol % [n(KI)]	
Electrolytes	Molar Ratio (KI:EG)
E1	1:5
E2	1:7
E3	1:9
E4	1:11

**Table 2:** Parameters extracted from Nyquist diagrams.

Electrolyte	$KI:EG:I_{2}$	$R_{s}(\Omega )$	$R_{ct}(\Omega )$	$R_{w}(\Omega )$	$Q_{O}$ $\left(s^{n}\Omega^{-1}\right)$	L(H)	(n)	χ^2^
E1	1:5	7.526±0.016	5.903±0.027	9.064±0.75	2.56±0.20	2.75±0.69	0.76±0.2	0.015
E2	1:7	5.612±0.035	3.054±0.053	7.296±0.22	25.79±0.33	2.36±0.49	0.83±0.2	0.017
E3	1:9	8.039±0.019	6.363±0.028	15.030±0.65	23.05±0.13	2.41±0.38	0.80±0.32	0.034
E4	1:11	7.613±0.033	6.768±0.029	21.59±0.42	21.58±0.25	2.78±0.58	0.68±0.13	0.002

## Data Availability

Data will be made available on request.
